# Editorial: Neural Circuitry of Behavioral Flexibility: Dopamine and Related Systems

**DOI:** 10.3389/fnbeh.2016.00006

**Published:** 2016-01-28

**Authors:** Gregory B. Bissonette, Matthew R. Roesch

**Affiliations:** ^1^Department of Psychology, University of MarylandCollege Park, MD, USA; ^2^Program in Neuroscience and Cognitive Science, University of MarylandCollege Park, MD, USA

**Keywords:** Dopamine, Pavlovian Instrumental Transfer, Sign and goal tracking, set shifting, endocannabinoid system, basal forebrain non-cholinergic neurons, motivational salience, ADHD

Dopamine neurotransmission has long been identified as a key component of prefrontal cortical function and flexible cognition; however, flexible behavior does not depend solely on dopamine. Together, multiple systems allow fast and flexible behavior in the face of a constantly changing environment. This Research Topic illuminates work by researchers who are leading efforts to understand how different neural systems yield flexible behavior.

The psychostimulant amphetamine is, paradoxically, a useful treatment for Attention Deficit Hyperactivity Disorder (ADHD) symptoms. Here, Yen and colleagues demonstrate in a mouse model of ADHD that the calming effect of amphetamine is due to decreased GSK3B, initiated through NMDA receptor signaling (Yen et al.).

Stress is known to be under the partial regulation of oxytocin, and oxytocin levels in adults may be influenced by severity of childhood maltreatment. The inconsistent quality of results on this topic are tackled by Mizuki and Fujiwara, who were able to demonstrate a correlation of severity of childhood maltreatment with diminishing oxytocin levels in humans (Mizuki and Fujiwara).

A key challenge in combatting drug addiction is the incentive motivational property of a stimulus. This is known to develop through Pavlovian Instrumental Transfer (PIT). Garofalo and di Pellegrino were able to show, for the first time in humans, that Sign-Tracker responses were biased by reward-paired cues while Goal-Trackers were not (Garofalo and di Pellegrino). This information is critical in developing individualized treatment plans for treating maladaptive behaviors.

Physical exercise is known to promote better cognition, but the mechanism remains unknown. In their article Berse and colleagues identified a particular single nucleotide polymorphism in the Dopamine Transporter (DAT1/SLCA6A3) gene which contributed to improved cognition under physical exercise conditions in adolescent humans (Berse et al.). These data suggest the usefulness in physical exercise to promote improved flexible cognition in adolescents.

Altered neurochemistry in the anterior cingulate cortex (ACC) is thought to be a root cause of the behavioral symptoms of ADHD. However, when Endres and colleagues conducted a large single voxel proton scale magnetic resonance spectroscopy study, they identified no neurometabolic differences in ACC or cerebellum between control and experimental groups (Endres et al.). These results fail to replicate an earlier and smaller experiment, and the authors propose that the difficulty in diagnosing ADHD may lie in a number of false-negative studies.

Flexible behavior is known to depend on the medial prefrontal cortex (mPFC). Bissonette and Roesch demonstrate how neural correlates of rules and conflict are encoded and signaled by mPFC neurons as rats modify their behavior in the face of changing contingencies (Bissonette and Roesch). Importantly, these populations of neurons also modified their activity based on feedback, significantly increasing activity for rewarded outcomes and significantly decreasing activity for non-rewarded choices. These results demonstrate how the mPFC signals the need for behavior to become flexible.

Raver and Lin present a very cogent review of the role that the basal forebrain plays in signaling arousal, attention and decision-making. They discuss relevant literature focusing on how a population of BF non-cholinergic neurons encode motivational salience through ensemble burst firing (Raver and Lin). This review serves focuses attention on how the BF salience system is a critical part of signaling attention towards relevant stimuli, promoting flexible behavior.

Most animal research uses reward-based learning paradigms, requiring animals to consume their reward. In this research topic, Parent and colleagues demonstrate how cholinergic and ghrelinergic signaling—both working through KCNQ channels in the mPFC—are critical for regulating the duration of licking(Parent et al.). These findings are important for interpreting data that manipulates the mPFC and uses reward-based learning paradigms.

Sign-tracking in animals is associated with increased sensitivity to food cues. Nasser and colleagues demonstrate that sign-tracking, but not goal-tracking, rats are more sensitive to food associated cues and that the degree of sign-tracking correlated with a failure to suppress responses during devaluation (Nasser et al.). These data suggest that natural variation among animals in their tendency towards either sign or goal-tracking may be correlated with drug addiction vulnerability.

The locus coeruleus (LC), which provides noradrenergic projects to the cortex, is important for attention. Janitzky and colleagues demonstrate that optogenetically suppressing LC activity during a set shifting task impaired performance on reversal and extra-dimensional set-shifting but not on learning of intra-dimensional shifts or compound discriminations (Janitzky et al.). These results suggest a role for LC not during initial learning, but during situations which require flexible shifts in behavior.

The lateral habenula (LHb) acts as a gatekeeper for information about negative outcomes being transmitted to the midbrain dopamine system. Baker and colleagues provide a thorough review of the LHb literature and present data to support a hypothesis whereby LHb neural activity is important for the maintenance of goal-directed actions to new response types during changing contingencies (Baker et al.). This information is necessary to guide future research on how LHb activity may be important for flexible behavior.

Particular behavioral responses need to be organized over time, allowing appropriate actions to occur at the right time. Parker, Ruggiero and Narayanan demonstrate that blockade of D1-type dopamine receptors in the medial frontal cortex leads to altered interval timing performance by impacting ramping patterns and differentially field potential activity without impacting baseline firing rates of neurons (Parker et al.). These data provide further support that frontal cortical neural activity works best with a basal level of dopamine.

Many neural systems interact while animals engage in complex behaviors and make decisions. Hernandez and Cheer provide a thorough review of the literature surrounding the endocannabinoid system and how endocannabinoid and dopamine systems may interact while animals make decisions (Hernandez and Cheer). This review summarizes the literature around how different aspects of the endocannabinoid system may modulate dopamine release.

Together, this collection of 13 works of original research and reviews provide both a new infusion of knowledge and a broad review of the neural systems underlying flexible behavior. Crossing a range of fields and using a diverse set of tools and subjects, this body of work has illuminated some exciting future avenues of research.

## Author contributions

GB and MR conceived of the Research Topic, edited manuscripts, and worked on this editorial.

## Funding

We would like to acknowledge our funder, National Institute on Drug Abuse (MRR DA031695; MRR DA040993).

### Conflict of interest statement

The authors declare that the research was conducted in the absence of any commercial or financial relationships that could be construed as a potential conflict of interest.

